# Antenatally detected urinary tract dilatation: long-term outcome

**DOI:** 10.1007/s00467-023-05907-z

**Published:** 2023-03-15

**Authors:** Maria Herthelius

**Affiliations:** 1 Astrid Lindgren Children’s Hospital, K88, Karolinska University Hospital, 141 86, Stockholm, Sweden; 2 Division of Paediatrics, Department of Clinical Science, Intervention, and Technology, Karolinska Institutet, Stockholm, Sweden

**Keywords:** Hydronephrosis, Urinary tract dilatation, Long-term outcome, Chronic kidney disease

## Abstract

This review provides updated knowledge on the long-term outcomes among children with antenatally diagnosed urinary tract dilatation (UTD), previously often referred to as antenatal hydronephrosis. Different definitions of UTD exist, which makes comparison between studies and generalized conclusions difficult. Roughly, one-third of antenatally diagnosed UTD, defined as a renal pelvis anterior posterior diameter (APD) of ≥ 4 mm in the second trimester and/or ≥ 7 mm in the third trimester, will resolve before birth, another third will resolve within the first years of life, and in the remaining cases, UTD will persist or a congenital abnormality (CAKUT) will be diagnosed postnatally. The risk of a postnatal CAKUT diagnosis increases with the degree of prenatal and postnatal dilatation, except for vesicoureteral reflux (VUR), which cannot be predicted from the degree of UTD. Urinary tract infections (UTIs) occur in 7–14% of children with UTD during the first years of life. The risk of UTI is higher in children with traditional risk factors for UTI, such as dilated VUR, hydroureteronephrosis, female gender, and intact foreskin. Continuous antibiotic prophylaxis may be considered in selected patients during the first years of life. In long-term follow-ups, permanent kidney damage is diagnosed in approximately 40% of children with moderate or severe UTD, but hypertension, proteinuria, and/or reduced eGFR are uncommon (0–5%). In children with mild UTD, the long-term outcome is excellent, and these children should not be subjected to unnecessary examinations and/or follow-up.

## Background

Antenatal urinary tract dilatation (UTD) is one of the most common fetal anomalies detected in pregnancy [1–5]. Previously, this condition was referred to as congenital, antenatal, or prenatal hydronephrosis, but UTD is now the preferred term.

The introduction of routine fetal ultrasound screening in the 1990s generated a largely undefined group of patients. As the significance of UTD was unclear, individual centers had to invent their own recommendations, which resulted in numerous suggestions for the definition of significant UTD and many different recommendations for monitoring and management. Knowledge increased over time, and we now know that most dilatations will eventually resolve without intervention; we refer to this as physiological or transient dilatation. However, in some cases, UTD may be the first indication of an underlying urinary tract anomaly. It is therefore necessary to find ways of identifying cases of significant uropathy without exposing children with physiological dilatation to unnecessary investigation and follow-up and without creating unnecessary concern among parents. This is one of the most challenging tasks for the obstetricians, radiologists, and pediatricians involved in the care of these patients.

The aim of this review is to provide updated knowledge on the long-term outcomes and management among children with antenatally diagnosed UTD. The underlying uropathies and their management have been extensively reviewed previously [6–8] and will therefore not be addressed here.

## Definition and grading of UTD

The aim of screening is to identify a specific condition and then initiate available treatment as early as possible, to avoid the risk of future morbidity. In UTD, this process includes identification of congenital anomalies of the kidney and urinary tract (CAKUT) and/or kidney damage. A prerequisite for successful screening is consensus over the definition of UTD, but it has not yet been possible to establish such a consensus, as cut-offs vary between studies and are often established arbitrarily. Some centers use the same cut-off values for both prenatal and postnatal evaluation, while others use increasing cut-offs for the different trimesters and for the postnatal evaluation.

The most common systems used for grading UTD are the APD system and the Society for Fetal Urology (SFU) system. However, other systems exist, such as the Onen system [9]. In the APD system, the anterior posterior diameter of the kidney pelvis is measured in millimeters. Different threshold values for abnormal dilatation have been used. For example, Dudley et al. proposed a cut-off of ≥ 5 mm throughout pregnancy [1], while Corteville et al. proposed ≥ 4 mm in the second trimester and ≥ 7 mm in the third trimester as definitions of significant pelvis dilation [10].

The SFU system uses a 5-point subjective grading which considers not only the appearance of the kidney pelvis but also the calyces and the kidney parenchyma [11]. However, the exact dimension of the kidney pelvis is not measured. An attempt to describe the SFU system with APD measures has been made by Swords and Peters [12].

A disadvantage of the grading systems mentioned above is that they do not provide information about the appearance of the ureters and bladder and the amount of amniotic fluid. Taking these measures into account is of utmost importance because a dilated bladder, a thickened bladder wall, dilated ureters, and oligohydramnios or anhydramnios may indicate lower urinary tract obstruction.

To address these shortcomings, a consensus conference was organized with the aim of improving the grading of UTD and harmonizing prenatal and postnatal UTD follow-up. Based on accumulated research, a new grading system and follow-up program was proposed [13]. The new system extracted important parts of the different pre-existing grading systems, adjusted limits for significant dilatation, and proposed a reduction in the number of follow-up investigations. Furthermore, it was suggested that the concept of hydronephrosis, which focuses only on the kidney, should be replaced by the concept of UTD, which emphasizes the importance of the entire urinary tract system. UTD is defined as mild, moderate, and severe based on APD measurements and other urinary tract findings (Table 1).

**Table 1:** Grading of UTD according to the UTD classification system

			Before birth			After birth
Old nomenclature		New nomenclature	Second trimester	Third trimester	New nomenclature	> 48 h
Mild		UTD A1	APD 4 to < 7 mm	APD 7 to < 10 mm	UTD P1	APD 10 to < 15 mm or central calyx dilatation
Moderate		UTD A2–3	APD ≥ 7 mm or abnormal kidney parenchyma, calyces, ureters, bladder, or amniotic fluid	APD ≥ 10 mm or abnormal kidney parenchyma, calyces, ureters, bladder, or amniotic fluid	UTD P2	APD ≥ 15 mm or peripheral calyx dilatation or ureter > 4 mm (with APD ≥ 10 mm or calyx dilatation)
Severe					UTD P3	Parenchymal abnormality, bladder abnormality and APD ≥ 10 mm or calyx dilatation

*UTD*, urinary tract dilatation; *APD*, anterior posterior diameter; *mm*, millimeters

Extensive postnatal investigation was proposed to be limited to those with moderate or severe dilatation. The UTD classification has been evaluated in several studies and has shown an accurate precision in detecting postnatal CAKUT, the need for surgery [14–16], and chronic kidney disease [17, 18]. Updated recommendations were published in 2022 [19].

## Postnatal evaluation

**Fig. 1:**
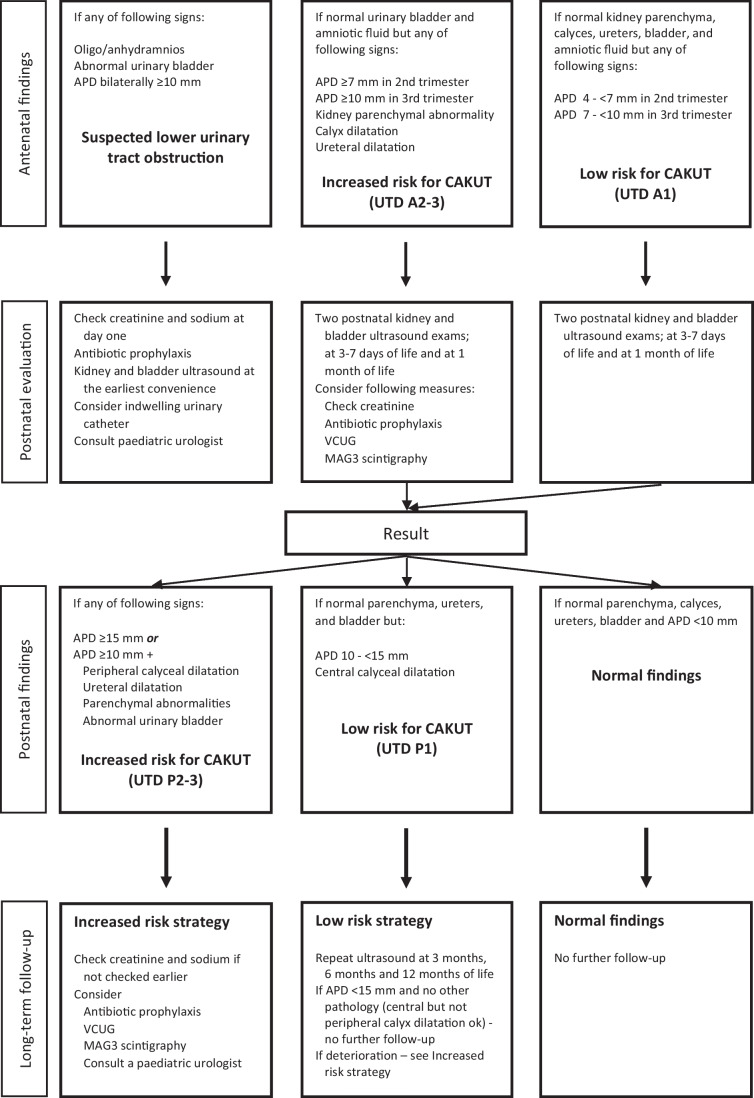
The Stockholm flowchart for antenatal and postnatal UTD evaluation. UTD, urinary tract dilatation; APD, anterior posterior diameter; CAKUT, congenital anomalies of the kidney and urinary tract; VCUG, voiding cystourethrogram; MAG3, mercaptoacetyltriglycine-3; mm, millimeters

Most protocols recommend that prenatal findings should be confirmed postnatally with two consecutive kidney ultrasounds [20]. Further management is based on the results of these investigations. The first ultrasound examination should, if possible, be postponed to at least 48 h after birth, because of the risk of a false-negative result due to low urine production during the first days of life. The second examination should take place a few weeks or months later, depending on the severity of the antenatal dilatation. A flowchart for both prenatal and postnatal care has been proposed by Nguyen et al. [13]. A similar flowchart used at the Karolinska University Hospital in Stockholm is presented in Fig. 1.

Postnatal investigation with invasive studies such as VCUG and MAG3 scintigraphy is usually reserved for children with postnatal APD ≥ 15 mm and/or abnormal kidney parenchyma, severe calyx dilatation, ureteral dilatation, or bladder pathology [13]. Children with APD 10 to < 15 mm and no other urinary tract abnormalities are usually monitored with repeated ultrasound examinations for some time to rule out progress of the dilatation. However, it has not yet been possible to define either the optimal period between the investigations or the proper time for discontinuation.

## What is the risk of a postnatal diagnosis of CAKUT?

Depending on the definition of UTD, CAKUT is diagnosed postnatally in approximately 25–50% of affected children [4, 21–23]. The risk increases with increasing degree of the antenatal dilatation [24] for all CAKUT conditions except vesicoureteral reflux (VUR), which is diagnosed in approximately 7–24% of all cases regardless of the degree of antenatal dilatation [4, 21, 22, 24–26]. A meta-analysis published in 2006 found that 12% of infants with mild dilatation, 45% with moderate dilatation, and 88% with severe fetal dilatation were postnatally diagnosed with CAKUT [24].

The most common CAKUT conditions diagnosed postnatally are VUR and ureteropelvic junction obstruction (UPJO), both diagnosed in approximately 10–12% of cases, followed by megaureter in 7% and different forms of kidney “plasias” (dysplasia, hypoplasia, and aplasia) in 6% [4, 22]. Conditions such as posterior urethral valve (PUV), prune belly syndrome, neurogenic bladder, ureterocele, and urethral aplasia are less frequently diagnosed. Although rare, it is particularly important to identify cases with possible lower urinary tract obstruction, for instance PUV, as the urinary tract may need decompression soon after birth to prevent further damage to the kidneys.

## What is the chance of spontaneous resolution of UTD?

The chance of spontaneous resolution depends on both the definition of UTD and whether all prenatally detected UTD is included or only UTD that persists into the postnatal period. It is important to separate data based on prenatal data from data based on postnatal data, as this knowledge is used to guide health care providers in the optimal surveillance of fetuses and infants with UTD and to provide information to the parents-to-be about what to expect in the future. Resolution in this context is used as a proxy for non-significant UTD, that is, a healthy child.

### Knowledge based on prenatal data

Most studies have used prenatal data and analyzed the chance of spontaneous resolution according to whether UTD is mild, moderate, or severe. Unfortunately, as mentioned above, definitions of mild, moderate, and severe UTD vary between studies. For example, Sairam et al. grouped their cohort of 268 fetuses with UTD according to findings in the second trimester; mild if APD was ≥ 4 mm and moderate/severe if APD was ≥ 7 mm or if there was calyceal dilatation. They reported a resolution rate of 82% for mild cases and 44% for moderate and severe cases [3]. Coelho et al. grouped their cohort of 192 fetuses with APD ≥ 5 mm, normal ureters and bladder, and normal amniotic fluid at gestational week 28 or later into mild (5 to < 10 mm), moderate (10 to < 15 mm), and severe (> 15 mm) and reported a resolution rate of 60% for mild UTD, 44% for moderate UTD, and 36% for severe UTD [23]. Barbosa et al. used a definition similar to Coelho et al. and reported a resolution rate of approximately 90% in mild UTD, 74% in moderate UTD, and 27% in severe UTD [25]. In the latter study, the addition of calyx dilatation to mild pelvis dilatation increased the risk of surgery from 9 to 14%; however, the authors did not state the indications for surgery.

Other research groups have concentrated on the subgroup of fetuses with dilated pelvis and/or calyces but normal kidney parenchyma, ureters, urinary bladder, and amount of amniotic fluid, often referred to as “isolated antenatal hydronephrosis.” For example, Tombesi et al. reported that resolution occurred in 73% of cases with isolated antenatal hydronephrosis before or after birth, and only 1% were subjected to surgery postnatally [27].

Mild prenatal UTD, mostly defined as an APD of ≤ 10 mm in the third trimester, often overlaps with the definition of isolated antenatal hydronephrosis. In some cases, however, APD may exceed this threshold without the co-existence of other urinary tract abnormalities, particularly in cases of UPJO. Nevertheless, regardless of the exact definition and grading of UTD, most studies show that a large proportion of antenatally detected UTD will resolve before birth [25–27] and a certain proportion more after birth and that the chance of resolution is higher in mild than in severe UTD [25].

### Knowledge based on postnatal data

Slightly more than 50% of cases that persist into the postnatal period will eventually resolve, and another 40–45% will improve or stabilize during the first 3 years of life [28–30]. Two studies with follow-up of at least 10 years found that resolution had occurred in 73–99% of cases with SFU grade I–II dilatation, 30–89% of cases with SFU III dilatation, and 0–31% of cases with SFU IV dilatation at the end of follow-up [31, 32]. Interestingly, Matsui et al. reported that the UTD reappeared in a small number of patients (1%) after being absent for several years [31].

Even though the chance of resolution in mild and moderate UTD is substantial, there is still a non-negligible risk of surgery in this group. Generally, the need for surgery is low, often reported to be between 0 and 2% [3, 21, 23, 33]. The indication for surgery is mainly VUR with recurrent urinary tract infections (UTIs) or UPJO with increasing dilatation and/or decreasing split function. Higher rates of surgery, such as the 10% reported by Barbosa et al. [25], possibly reflect a different policy for surgery.

## What is the risk of urinary tract infections?

Children with antenatally diagnosed UTD have an increased risk of UTI compared to the age-matched normal population. Most studies report a cumulative incidence of approximately 7–14% during the first 3 years of life [21, 23, 27, 28, 33–38], but inclusion criteria vary between studies, which makes comparisons difficult. For example, Szymanski et al., Zareba et al., Zee et al., and Visuri et al. excluded children with anatomical abnormalities other than VUR [34–37]; Tombesi and Alconcher, Madden-Fuentes et al., and Alconcher and Tombesi excluded children with anatomical abnormalities as well as those with VUR [27, 28, 33]; Coelho et al. excluded children with abnormal amniotic fluid and/or bladder or ureter dilatation in utero [23]; and de Kort and Lidefelt did not exclude any cases [21, 38]. Single studies have reported a lower UTI frequency than the abovementioned [39], while for example Braga et al., who unlike most others included patients and collected data prospectively, reported a UTI frequency of 19% [40]. The prospective design may have contributed to the higher cumulative incidence in this study.

Several studies have attempted to define risk factors for UTI within the UTD population. Coelho et al. confirmed that underlying uropathy was an independent risk factor for febrile UTI [41], and in the study by Visuri et al., VUR seemed to be the most important risk factor among the uropathies [37]. Braga et al. concluded that hydroureteronephrosis, female gender, an intact foreskin, and no antibiotic prophylaxis were risk factors for UTI [40]. However, Lidefelt et al. showed that the risk of UTI in children with mild UTD was similar to the age-matched population and therefore did not recommend antibiotic prophylaxis for this group [38]. Moderate/severe UTD (SFU grade III–IV) as opposed to mild UTD (SFU grade I–II) seems to be an independent risk factor only in boys [36].

## What is the risk of permanent kidney damage?

Perhaps the most important question in the long term is who will end up with chronic kidney damage later in life and who will not. Children who already exhibit kidney damage at birth, such as those with congenital kidney dysplasia or hypoplasia, naturally belong to this group. However, in an unselected group of children with antenatally detected UTD, it is challenging to predict who else will end up with permanent kidney damage. This question has been addressed by only a few research groups.

The Oliveira group from Brazil is one of these [42]. They analyzed a cohort of 447 children with isolated antenatal APD ≥ 5 mm in the third trimester and showed that 5% of the children had experienced a composite event of hypertension, proteinuria, and/or reduced eGFR at a median follow-up of 6.4 years (IQR: 2.8–12.5 years). All children who experienced this composite event were defined as SFU III–IV, and so, no child with mild UTD developed chronic kidney damage during the study period [42].

Herthelius et al. studied a smaller group of children diagnosed with UTD at a single center in Stockholm between 2003 and 2005 [43]. Approximately two-thirds of the original cohort (71/103, 69%) agreed to a follow-up at an average age of 13.6 years (range: 12–15 years). None of the participants in this study exhibited proteinuria or reduced eGFR at follow-up. However, among children with postnatal APD > 7 mm and/or kidney, calyceal, ureteral, or bladder pathology in the neonatal period, 15% had persistent UTD and 39% had signs of permanent kidney damage (according to either ultrasound or DMSA findings) at the 12–15-year follow-up. Among children without these findings, only one boy (5%) had signs of chronic kidney damage. Among children with chronic kidney damage at the 12–15-year follow-up, all but one (7%) had moderate or severe antenatal UTD (UTD A2–3).

## At what time point can follow-up safely be stopped in a child who has dilatation postnatally that is neither a basis for further investigation nor small enough to be ignored?

At the present time we have no definite answer to this question. What we do know is that with today’s recommendations, we will miss sporadic cases with minor urinary tract abnormalities and/or minor kidney damage, for instance VUR. This is unavoidable without exposing a large number of healthy children to unnecessary investigations.

The follow-up of children diagnosed with CAKUT should be individualized and based on recommendations from a pediatric nephrologist, a pediatric urologist, or both. Children older than 1 year and with an APD less than 15 mm and no other abnormal findings on repeated measurements are unlikely to be diagnosed with CAKUT later in life and can be discharged after informing the parents to seek medical advice in case of recurrent UTIs or recurrent or persistent abdominal pain [6, 44].

## Summary and conclusions

Roughly speaking, one-third of prenatally detected UTD will resolve before birth, another third will persist into the neonatal period but resolve spontaneously within the first 2 to 3 years of life, and the remaining third will end up with a CAKUT diagnosis in the early neonatal period or with persistent UTD.

In children with CAKUT, chronic kidney damage may be present at birth or may develop later. This group of children should be monitored for kidney damage during childhood.

In children with no CAKUT diagnosis but with persistent moderate or severe UTD (UTD P2–P3, SFU III–IV), there is a small but non-negligible risk of future kidney damage. This group could include cases with undiagnosed CAKUT, mostly VUR or low-grade UPJO, and the optimal follow-up is unclear. It may therefore be wise to infrequently monitor these children during childhood and to carefully instruct the parents to seek medical advice in case of recurrent UTIs or recurrent or persistent abdominal pain.
